# Body-Related Attentional Bias among Men with High and Low Muscularity Dissatisfaction

**DOI:** 10.3390/jcm9061736

**Published:** 2020-06-04

**Authors:** Bruno Porras-Garcia, Erik Exposito-Sanz, Marta Ferrer-Garcia, Oscar Castillero-Mimenza, José Gutiérrez-Maldonado

**Affiliations:** Department of Clinical Psychology and Psychobiology, University of Barcelona, Passeig de la Vall d’Hebron 171, 08035 Barcelona, Spain; brporras@ub.edu (B.P.-G.); erikexpositosanz@hotmail.com (E.E.-S.); martaferrerg@ub.edu (M.F.-G.); oscar_bcn_1991@hotmail.com (O.C.-M.)

**Keywords:** body-related attentional bias, muscularity dissatisfaction, men, virtual reality, eye-tracking

## Abstract

Previous studies have found gender differences in body-related attentional bias (AB), with women showing AB towards weight-related body parts. However, few studies have assessed the relationship between body-related AB and muscularity dissatisfaction (MD) in men. This study aimed to assess the presence of muscle-related AB in men, using a combination of a virtual reality (VR) embodiment-based technique and eye-tracking (ET) technology. Twenty men with high MD and 20 with low MD, owned a virtual avatar that had the same silhouette and body mass index as the participant. To analyze the gaze data, muscle-related areas of interest (M-AOIs) and nonmuscle-related areas of interest (NM-AOIs) were defined. The complete fixation time and the number of fixations on each AOI were recorded. Mixed between (group)-within (AOI_condition) analyses of variance showed a statistically significant interaction between group and time (*p* < 0.05) in both AB measures. Follow-up analyses revealed an AB towards M-AOIs only in men with high MD. Overall, men with high MD spent more time looking and displayed a higher number of fixations on M-AOIs, specifically the chest and shoulders, compared to men with low MD. This study provides new information about the relationship between MD and body-related AB in men. Combining VR with ET technologies presents interesting opportunities in the study of body image in men.

## 1. Introduction

Body image studies have been typically conducted in women, with fewer studies focusing on men. In fact, research on male body image is considered a relatively recent field of study [1], with important gaps, such as those in research on male body image and eating disorders [2]. Body image has been described as a multidimensional construct reflecting the mental representation a person has of their physical appearance [3], which includes perceptual, cognitive, attitudinal, and affective components [4]. Individuals with body image dissatisfaction usually experience negative and dysfunctional cognitions and emotions (sadness, anger, or disgust) related to the way they perceive, evaluate, and feel about their own body [1,5]. Among adult men, body image dissatisfaction is common phenomenon, with a prevalence ranging from 9% to 28% [6] and 8 to 64% [7] in the US population.

There is consensus that body dissatisfaction in men is mainly associated with two central factors, muscularity and body fat [8,9]. For instance, the ideal male body usually presented in Western media is that of a lean muscular body with well-developed chest, arms, shoulders, and legs [10,11], with the internalization of the ideal muscular body as a mediating factor for concerns associated with both muscularity and body fat [12]. Previous studies have found that the pathological drive for muscularity and body weight concerns among men are related to greater depressive symptomatology, low self-esteem, more physical exercise, and abuse of anabolic-androgenic steroids [10,13,14]. Likewise, persistent and intense concerns about the lack of muscularity and/or excessive body fat might lead to body image disorders, such as muscle dysmorphia [15].

According to the DSM-5 [16], individuals with muscle dysmorphia usually experience a pathological preoccupation with the idea that his or her body build is too small or insufficiently muscular. Other studies have suggested that individuals with a high risk of developing muscle dysmorphia might share some of the psychopathological symptoms of individuals with anorexia nervosa (AN), such as a negative affective state, body image disorders, more physical exercise, and significant avoidance or negative body checking strategies [17,18]. For instance, previous studies suggest that young men with symptoms of muscle dysmorphia reported similar body-checking behaviors than individuals with eating disorders [19]. This suggests an implicit tendency to pay more attention to certain types of information (e.g., disorder-relevant information) over others, which is also referred to as attentional bias (AB) [20]. Dysfunctional body-related AB presumably maintains body dissatisfaction by processing negative information that is consistent with dysfunctional cognitive schemas (such as *I am getting a fatter belly* or *my arms are not muscular enough*), while schema-inconsistent information is not noticed or processed equally, usually being neglected visually [21]. Accordingly, these dysfunctional cognitive schemas and body-related AB elicit a negative affective state and compensatory or avoidance behaviors [20].

Previous studies have found that women with eating disorders (ED) and individuals with high levels of body dissatisfaction show AB towards “disorder-salient stimuli,” including thin bodies, weight-related body areas, or self-reported unattractive body areas [22,23,24,25].

According to those studies that assess body-related AB among men, two main paradigms have been proposed. In the first paradigm, different competing stimuli (usually bodies with different shapes) are presented at the same time. Previous studies using well-established AB methods, such as the dot probe task and eye tracking (ET) technology, have observed specific body-related AB towards idealized thin and muscular bodies over other types of body sizes or neutral images in men with high body dissatisfaction [26,27]. Similar results were found among men at high risk of developing muscle dysmorphia after conducting a visual probe task [28].

In the second paradigm, participants are presented single images of their own or other types of bodies, during which attentional patterns towards specific body areas are measured. Two previous studies conducted with ET technology found preliminary evidence of AB towards weight-related body areas among men with a high drive for thinness [29] and AB towards upper body areas (e.g., chest, shoulders, and abdomen) in male college students when exposed to photographs of attractive males [30]. Comparing self-reported attractive vs. unattractive body parts, Cordes, Vocks, Düsing, Bauer, and Waldorf [31] reported that male weight trainers showed greater AB, as assessed by ET technology, towards their self-reported attractive body areas when exposed to pictures of a muscular athletic body. Additionally, a recent ET study found that men with muscle dysmorphia showed AB towards their self-reported unattractive body parts when looking at pictures of their own bodies [32]. 

In summary, results on body-related AB in men indicate that there was AB towards muscular bodies when bodies with different shapes were presented. There is also evidence suggesting that men with muscle dysmorphia show AB towards self-reported unattractive body parts [32].

In the field of body image and body-related AB, several methodologies have been used to measure different attentional processes. Some of these methodologies are based on reaction-time measures, such as the Stroop tasks, dot-probe tasks, and visual search tasks. The Stroop tasks are usually employed to measure attentional maintenance and avoidance, while the dot-probe or visual search tasks are used to distinguish between attentional mechanisms (e.g., attentional maintenance and avoidance mechanisms) [33]. However, some of these reaction time measures might not be equally useful in assessing change or dynamic attentional patterns during a certain amount of time [33,34]. To do so, ET technology has been recommended, which can provide a continuous and dynamic measure of AB. In fact, all the studies mentioned above (e.g., [29,31,32]) used fixed ET devices.

Although ET technology is a useful tool for assessing body-related AB in healthy and clinical samples with eating disorders [35], it also presents some important limitations, such as the lack of external validity [36]. The use of virtual reality (VR) technology may provide interesting new insights into the assessment of body-related AB, as well as help to overcome this drawback, by adding ET devices into a head-mounted display (HMD). For instance, it provides researchers with an accurate, objective and real-time measurement of an individual’s gaze patterns while participants are fully immersed in a VR scenario that represents a real-life situation [37]. In addition, based on the rubber hand illusion paradigm [38], the use of an embodiment-based technique induces the illusion of ownership of a virtual body (i.e., participants regard the virtual body as their own). This avatar may even simulate a real-size 3D simulation of the body of an individual with their specific physical features [39]. Finally, the use of VR embodiment-based techniques has already shown promising results in changing dysfunctional body representations in individuals with AN [40,41] and in healthy participants [42,43,44], in a paradigm recently referred to as embodied medicine [45].

A recent study assessed gender differences in body-related AB using an ET and VR embodiment-based technique. Participants were first exposed to an avatar with the same measurements as their own bodies, then to a larger-sized virtual body before being exposed to the first virtual body [46]. The authors found that women showed AB towards weight-related body parts, while men showed AB towards muscle-related body parts. Body dissatisfaction levels among men and women did not significantly affect the results [46].

The current study aimed to provide further information about the relationship between body-related attention and muscularity dissatisfaction (MD) among men. Specifically, body-related attention towards particular muscle- or nonmuscle-related body parts were assessed in men with high vs. low levels of MD who were exposed to a virtual avatar that had their real silhouette and body mass index (BMI).

To the best of our knowledge, this is the first study assessing body-related AB and comparing muscle- to nonmuscle-related areas of interest (AOIs) in men, in contrast to other studies using different methodologies to compare groups of AOIs (e.g., self-reported unattractive vs. attractive AOIs). Additionally, this study presents an innovative assessment procedure in which VR and ET technologies were combined to assess body-related AB in men. Based on the previous research [32,46], it was expected that all men, regardless of their MD levels, would show AB towards muscle-related body areas. Furthermore, men with high MD were expected to show greater AB than those with low MD.

## 2. Materials and Methods

### 2.1. Participants

Forty male college students from the University of Barcelona, comprising 20 men with low MD (*M*_age_ = 24.65, *SD* = 3.94, *M*_BMI_ = 24.84, *SD* = 1.98) and 20 men with high MD (*M*_age_ = 24.05, *SD* = 3.31, *M*_BMI_ = 23.71, *SD* = 2.30), participated in the study. All participants were voluntarily recruited through campus flyers or by direct contact. The exclusion criteria were: a BMI of less than 17 (moderate thinness) or more than 30 (obesity; according to the World Health Organization [47]), a self-reported diagnosis of current muscle dysmorphia or an eating disorder, and other self-reported current severe mental disorders (e.g., schizophrenia or bipolar disorder). Visual deficits that impeded VR exposure, epilepsy, and clinical cardiac arrhythmia were also criteria for exclusion.

### 2.2. Measures

#### 2.2.1. Visual Selective Attentional Bias Measures 

**Figure 1:**
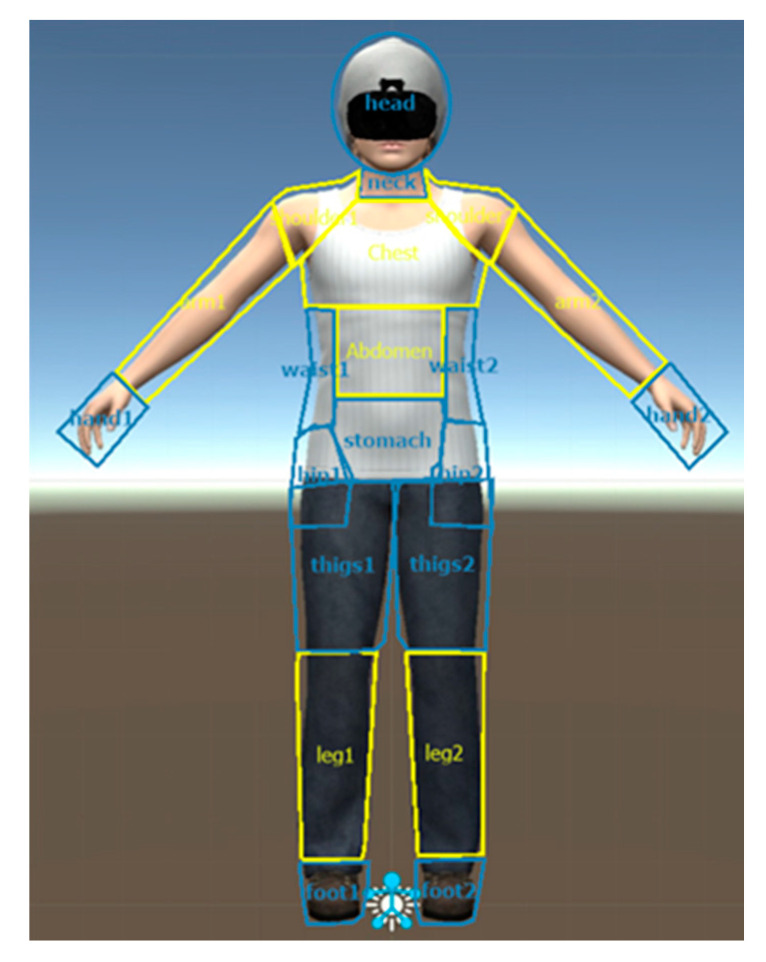
Muscle-related and nonmuscle-related areas of interest on the male virtual avatar.

In accordance with the body items loading for muscularity of the Male Body Attitudes Scale (MBAS; 8) and the Male Body Checking Questionnaire (MBCQ) [48], the same AOIs were individually drawn onto a 2D frontal view image of the male avatar and were labeled as muscle-related body parts (M-AOIs), i.e., the chest, arms, shoulders, abdomen, and lower legs. The remaining body areas were labeled as nonmuscle-related body parts (NM-AOIs), i.e., head, neck, hands, stomach, hips, waist, thighs, and feet (Figure 1). The same M-AOI and NM-AOI labels were used in a previous study conducted by our group [46].

The term fixation is defined as the act of sustaining one’s gaze on a single location for a minimum amount of time, typically 100–200 ms [49]. The following measures were used to assess AB:

- *Number of fixations on AOIs*: Number of fixations on a specified group of AOIs (M-AOIs or NM-AOIs).
- *Complete fixation time on AOIs*: Sum of the durations of the fixations on a specified group of AOIs (M-AOIs or NM-AOIs) in milliseconds.

#### 2.2.2. Assessment of Muscularity Dissatisfaction

*Male Body Attitudes Scale* [8] is a self-report questionnaire that assesses male body image. It includes 3 scales (muscularity dissatisfaction, pursuit of low body fat and height) and 24 items that are scored on a 6-point Likert scale, ranging from 1 (*never*) to 6 (*always*). After a confirmatory factor analysis, the Spanish version of this questionnaire was developed, which includes 22 items after excluding the 2 items of the height scale [50]. For the purposes of this study, only the muscularity subscale (MBAS-M) was administered, which contains 10 items. Higher average scores indicate greater MD, with scores ranging from 1 to 6. Both the original and Spanish version of this questionnaire presents robust validity indices and good reliability, with Cronbach’s alpha ranging from 0.85 to 0.90 [50]. Cronbach’s alpha for the MBAS-M scale in the current study was 0.87.

### 2.3. Hardware and Software Features

All participants were exposed to an immersive virtual scenario through a VR head-mounted display (HMD HTC VIVE-Pro, HTC Corporation, New Taipei City, Taiwan). In addition, two HTC VIVE-Pro controllers and three additional body trackers were used to achieve full-body motion tracking. VR body trackers allow the movement of objects or people within the virtual environment with high fidelity. HMD HTC VIVE-Pro has dual OLED panels at a resolution of 1440 × 1600 pixels per eye (2880 × 1600 combined), with a refresh rate of 90 Hz. It also has 110° vision and is easily adaptable to facial features. The entire VR equipment was connected to a VR-ready computer with a powerful processor (*Intel Core* i7-8700k with 3.70 GHz, Intel Corporation, Santa Clara, CA, USA) and a graphic card (Nvidia RTX 2080, Nvidia Corporation, Santa Clara, CA, USA) to run the VR environments.

The VR HMD FOVE Eye Tracking (FOVE, Inc., Torrance, CA, USA) was used to detect and register eye movements. The headset uses incorporated position and orientation eye-tracking systems. The FOVE display has a resolution of 2560 × 1440 pixels and creates 70 frames per second. Infrared eye tracking sensors create 120 frames per second, with an accuracy level of less than 1°.

FOVE Setup v0.16.0 (FOVE, Inc., Torrance, CA, USA) and Unity 3D 2018 v2.10 (Unity Technologies, San Francisco, CA, USA) were used to create virtual simulations. Unity 3D was used to develop the object-oriented programming code and integrate the elements within a virtual environment. Virtual avatars were created using the software Blender 2.78 and Gimp for improved texturing. The virtual environment was a simple room without any furnishings, but with a large mirror on the front wall that was placed 1.5 m in front of the avatar. The room also had a slightly open door to avoid possible feelings of being locked in. The avatar represented a young male, wearing a tight white t-shirt, blue jeans, and a swimming cap to avoid any influence of hairstyle.

### 2.4. Procedure

This study was approved by the ethics committee of the University of Barcelona. Participants had to provide informed consent before entering the study. Each participant was informed of the procedure and about data confidentiality and was told that they could withdraw from the study at any point without consequences. Confidentiality was ensured by assigning a different identification code.

Each participant was weighed and measured to calculate their BMI. Additionally, the researchers interviewed the participants to ensure that they did not meet the exclusion criteria.

**Figure 2:**
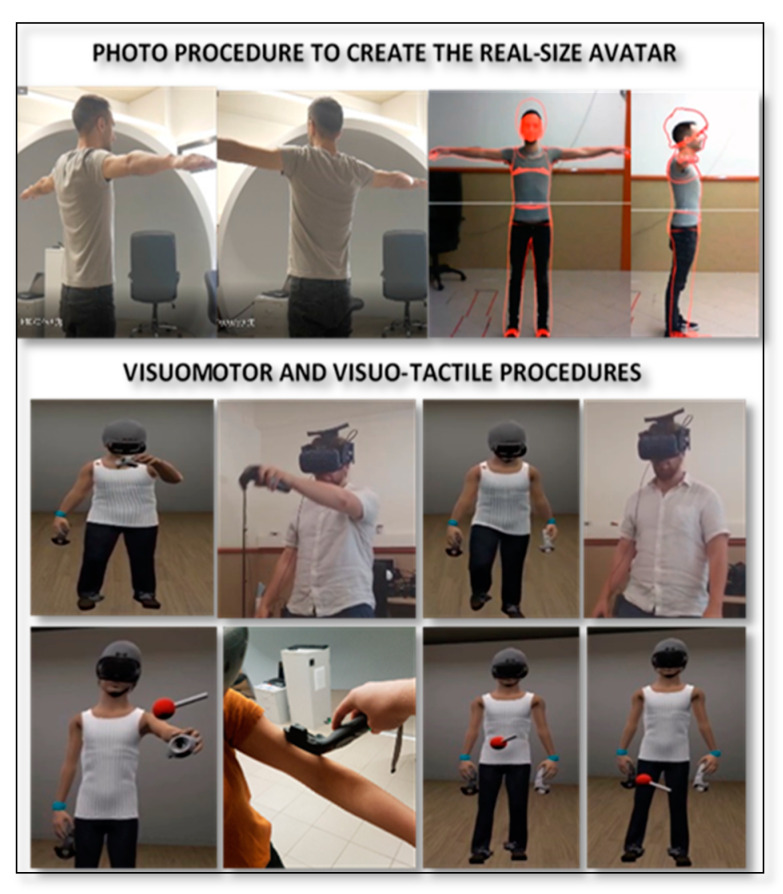
Illustration of the procedures used to induce full-body illusion in the study.

To create the avatar, a frontal and lateral photo of the whole body of the participant was taken using a camera. Each participant was placed at a fixed position 2 m from the camera and was told to stand still with their arms slightly raised and legs slightly apart. The BMI of the participant was manually inputted into the software, while the body size of the avatar was automatically adjusted based on the BMI value. The participant’s photo and virtual silhouette were then matched by manually adjusting (e.g., increasing or decreasing) the different parts of the virtual silhouette (shoulders, arms, chest, waist, stomach, hip, thighs, and legs) to fit the silhouette of the participant (for an illustrative example, see Figure 2). In the meantime, the Muscularity subscale (MBAS-M) of the MBAS was administered.

Participants were exposed to the VR environment through the HMD HTC VIVE-Pro. Once inside the virtual environment, each participant was able to observe himself in the first-person perspective and to look at himself in the mirror (in the third-person perspective). Full-body illusion (FBI) with the virtual body was induced using visual-motor and visual–tactile stimulation (for an illustration, see Figure 2). The visual-motor stimulation procedure was adapted from study of Waltemate, Gall, Roth, Botsch, and Latoschik [51] and consisted of synchronizing the movement of the participant with that of the avatar using motion capture sensors placed on the hands, feet, and waist. The visual–tactile stimulation procedure consisted of synchronizing visual and tactile stimulations. While the different areas of the body (upper and lower limbs and stomach) were touched on the participant, each participant observed the same areas being touched on the avatar at the same time, which was performed by a virtual controller. The visual–tactile procedure was adapted from previous studies on embodiment involving the abdomen [40] or the whole body [46]. Both procedures lasted for 3 min.

Finally, to assess body-related AB, the HMD was replaced with the VR HMD FOVE Eye Tracking headset. The virtual room displayed on the HMD FOVE was the same as that in the previous VR environment, with the participant’s real-size avatar reflected in the mirror.

First, the accuracy of the ET recordings was measured using a 9-point calibration procedure. The individual’s gaze was then tracked while they were asked to observe their real-size virtual body in the mirror for 30 s, a similar recording time to that used in previous studies [23,24]. Throughout this process, the participant was instructed to avoid abrupt head movements. During the process and as a cover story, the participant was told to remain still while the position of the avatar was being recalibrated.

### 2.5. Statistical Analysis

The OGAMA (Open Gaze and Mouse Analyzer) software was used to transform the ET raw data into suitable quantitative data. Complete fixation time and the number of fixations on the AOIs were calculated for each participant. This was achieved by summing up the separate complete fixation times and the number of fixations on the M-AOIs vs. on the NM-AOIs. An additional data transformation was conducted by calculating the difference between M-AOIs and NM-AOIs (e.g., for the complete fixation time, 2510 M-AOIs − 2110 NM-AOIs = 400). Therefore, a positive outcome indicated that the participant had been looking more at the muscle-related body parts than at the nonmuscle-related body parts, while a negative outcome indicated the opposite.

Statistical analyses were conducted with the statistical software IBM SPSS Statistics v.24. An a priori alpha level of 0.05 was considered for the study.

All the participants were divided into two groups: one with high MD and the other with low MD. Since the original test did not provide a standardized cut-off score for dividing the groups [8], and since other methods for splitting the sample (e.g., recruiting the top and bottom thirds) would have notably reduced the sample size, it was decided to use the median score of the MBAS-M (M_e_−MBAS-M = 3) as a cut-off score for this study.

Mixed between (group)-within (M-vs-NM AOIs condition) analyses of variance (ANOVA) were conducted with both body-related AB measures (complete fixation time and number of fixations).

Furthermore, independent-sample *t* tests were run to determine if there were significant differences in body-related AB between men with high and low levels of MD. These analyses were conducted for the groups of AOIs (e.g., difference between M-AOIs and NM-AOIs) and for single M-AOIs (e.g., arms, shoulders, chest, etc.).

Finally, Pearson’s correlations analyses were conducted to assess the relationship between BMI and body-related AB measures separately for men with high and low MD.

All assumptions required for two-way mixed ANOVAs were met. There were no outliers in the data, as determined by the inspection of a boxplot. Scores for both ET measures for each group were normally distributed as determined by the Shapiro–Wilk test (*p* > 0.05), and there was homogeneity of variances as assessed by Levene’s test for equality of variances (*p >* 0.05). Regarding the assumptions required for independent-sample *t* tests, nonparametric Mann–Whitney U tests were used in the analyses conducted with the single M-AOIs, since data for some of the variables were not normally distributed.

## 3. Results

The mean MBAS-M score was 2.23 (*SD* = 0.39) among men with low MD and 3.66 (*SD* = 0.60) among those with high MD. Group differences in age, BMI, and muscularity dissatisfaction levels were assessed using independent *t* test analyses. Results showed that the groups did not differ significantly on measures such as age and BMI (*p* > 0.05), but did differ significantly in muscularity dissatisfaction levels (*p* < 0.001). The mean and standard deviations of the two ET measures are shown in Table 1.

Two-way mixed ANOVA showed statistically significant interactions between the group and AOI_condition on complete fixation time (*F* (1, 38) = 8953, *p* = 0.005, partial *η^2^* = 0.191), and number of fixations (*F* (1, 38) = 6275, *p* = 0.017, partial *η^2^* = 0.142). Follow-up analyses were conducted, and mean differences (*MD*) ± standard error (*SE*) are specified. These analyses showed that men with high MD spent significantly more time looking at M-AOIs and presented a significantly higher number of fixations on M-AOIs than on NM-AOIs (complete fixation time, *MD* = 5.750 ± *SE* = 1.923, *p* = 0.005, *d* = 0.65 and number of fixations, *MD* = 9.50 ± *SE* = 2.40, *p* < 0.001, *d* = 0.82). On the other hand, men with low MD did not show significant differences in AB between M-AOIs and NM-AOIs (complete fixation time, *MD =* 2.387 *± SE* = 1.923, *p* = 0.222, *d* = 0.28 and number of fixations, *MD* = 1.00 ± *SE* = 2.40, *p* = 0.679, *d* = 0.10). Overall, they spent a similar amount of time on M-AOIs and NM-AOIs and presented an equal number of fixations on the two types of areas.

**Figure 3:**
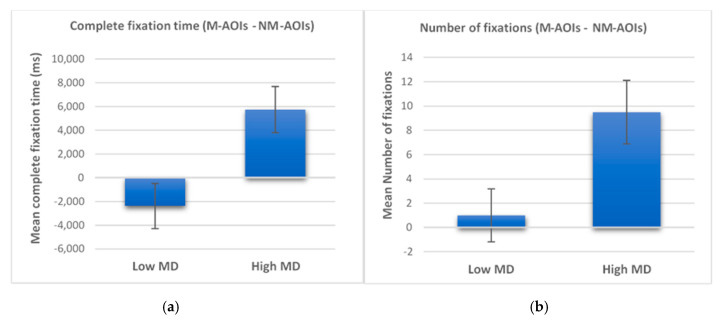
Differences between men with high and low muscularity dissatisfaction (MD) in mean complete fixation time (in milliseconds; ms) and mean number of fixations on muscle (M)-related vs. nonmuscle (NM)-related areas of interest (AOIs). Error bars represent standard error of the mean (SEM). (**a**) Complete fixation time; (**b**) Number of fixations.

Furthermore, considering differences between M-AOIs and NM-AOIs, men with high MD spent more time looking at M-AOIs and displayed a higher number of fixations on M-AOIs compared to individuals with low MD (see Figure 3). Independent-sample *t* tests revealed that these group differences were significant (complete fixation time, *t* (38) = 2.992, *p* < 0.01, *d =* 0.95 and number of fixations, *t* (38) = 2.005, *p* = 0.01, *d* = 0.79).

**Figure 4:**
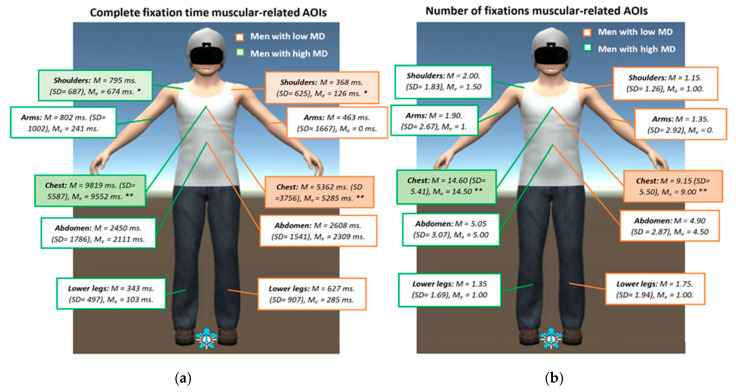
Differences between men with high and low muscularity dissatisfaction (MD) (in mean, standard deviation, and median) in complete fixation time (in milliseconds; ms) and number of fixations on individual muscle-related areas of interest (AOIs). Mann–Whitney U test analyses: * *p* = 0.04, ** *p* < 0.01. (**a**) Complete fixation time; (**b**) Number of fixations.

Mann–Whitney U tests were run to assess group differences in AB measures in each single M-AOIs. Distributions of the complete fixation time and the number of fixations were similar for men with high and low MD, as determined by visual inspection. The median for complete fixation time and number of fixations was significantly higher in men with high MD than in those with low MD for the chest (complete fixation time, *U* = 302, *z* = 2.769, *p* = 0.005 and number of fixations, *U* = 301, *z* = 2.741, *p* = 0.006) and shoulders (complete fixation time, *U* = 272, *z* = −2.004, *p* = 0.045), using an exact sampling distribution for U. Overall, men with high MD spent more time looking at the upper body areas (arms, shoulders, and chest), while both groups spent an equal amount of time looking at the abdomen and the lower legs (see Figure 4).

Finally, Pearson’s correlations analyses were conducted to assess the relationship between BMI and body-related AB measures separately for each group. There were no significant relationships between BMI and complete fixation time (*p* > 0.05) either in men with high MD or in men with low MD. In addition, there was no significant relationship (*p* > 0.05) between BMI and number of fixations in men with low MD. However, there was a statistically significant, moderately negative correlation between BMI and number of fixations (*r* (18) = −0.50, *p* = 0.024) in men with high MD, with BMI explaining 25% of the variation in number of fixations. This means that, among men with high MD, those with higher BMI values looked at M-AOIs less frequently.

## 4. Discussion

This study aimed to assess body-related AB towards specific muscle-related or nonmuscle-related body parts in men with high vs. low MD. We also present a new body-related AB assessment methodology that involves VR and ET technologies. In contrast to previous studies measuring gaze behavioral patterns in individuals looking at a picture or a photograph of their own body, we exposed participants to a virtual avatar with their real silhouette and BMI in a virtual room with a large mirror in front of them. As expected, men with high MD spent more time and looked more frequently at muscle-related body areas, especially the shoulders and chest, than those with low MD. Additionally, AB towards specific muscle-related body areas was found only among individuals with high MD.

Consistent with the findings of Porras-Garcia, Ferrer-Garcia, Ghita et al. [46] that there is AB towards muscle-related body parts in men, our results provide further evidence about the relationship between body-related AB and MD. Although both studies used a similar assessment procedure with VR embodiment-based techniques and ET devices, there are some key differences between the studies. The current study found significant differences in body-related AB between men with high and low MD, which is in contrast to the previous study that did not find such AB differences between men with high and low body dissatisfaction. Although body dissatisfaction is certainly associated with muscularity in men [9], there are other body factors (e.g., concerns with body fat or slimness [9,10,11]) that might also influence male body image. Consequently, differences in body-related AB towards muscle-related body areas might be easier to detect between men with high vs. low MD than between men with high vs. low body dissatisfaction. Another important difference between the two studies was the technical improvements made to the software used in this study, such as developing a more precise method to create the avatar (by taking a frontal and lateral photo and incorporating the information of the BMI of each participant) and improving the full-body tracking system to conduct visual-motor stimulation.

Our results also support those of previous studies by providing further evidence of AB towards muscle-related body parts in men with a high drive for muscularity [28,32]. However, there are several methodological differences between the current study and others in this field. For instance, as described before, some of the studies on men with high body dissatisfaction [26] or at high risk of developing muscle dysmorphia [28] assessed AB for competing stimuli, such as different body shapes (e.g., muscular bodies vs. normal or overweight bodies), while others (e.g., [31,32]) used a similar free-viewing single-body paradigm but measured AB towards self-reported attractive vs. unattractive body areas. Although these previous studies used a successful and well-established methodology to define areas of interest [21,35], evidence for AB towards specific muscle-related body areas could not be clearly established, since there were muscle-related body areas in both the self-reported attractive and unattractive body parts [31,32]. In this study and in two previous studies, a different methodology was used to define areas of interest, in which an individual’s gaze behavior was analyzed using the same definition of areas of interest for all participants (e.g., weight- vs. nonweight-related body areas [46,50]). These areas of interest were defined based on well-established questionnaires that assess the same body image construct, such as the Muscularity subscale of the MBAS [8] and the MBCQ [48].

In men with low MD, differences in AB measures towards muscle-related and nonmuscle-related body parts did not reach significance, suggesting that they generally scanned over the whole body compared to men with high MD. This is in line with previous research reporting a similar behavior among healthy women [22,23,25] and healthy men [28,46].

Regarding specific muscle-related body areas, we observed that, regardless of the MD group, all men paid more attention to the chest and abdomen. These results support previous findings reported by Cordes et al. [31] in which a similar attentional behavior was reported among men towards the same two body areas. Moreover, there were significant differences in AB towards the chest between men with high and low MD for both AB measures, while the same was observed for AB towards the shoulders, but only for the complete fixation time. This suggests that these two body areas may be particularly salient for men with high MD. Surprisingly, there were no significant group differences for other muscle-related body areas such as the abdomen, arms and lower legs, indicating that the two groups spent similar amounts of time looking at these specific body parts. This could be because these three body areas might be related not only to muscularity but also to body fat. As mentioned before, male body dissatisfaction is associated with two central factors, muscularity and body fat concerns [8,9]. Thus, paying attention to these body areas might not necessarily imply AB towards muscularity, but might be influenced by other body image concerns as well. The high prevalence of body dissatisfaction among healthy men [6,7] might also explain why men with high and low MD displayed similar gaze patterns towards these specific body areas. Finally, preliminary evidence was found suggesting that, among men with high MD, those with higher BMI values looked at M-AOIs less frequently. Even though these results were found in only one of the two AB measures (number of fixations), they may suggest a distinct visual attentional pattern toward the body between men with high MD and high BMI compared with men with high MD but within a normal BMI range. However, the results of the current study do not allow us to state conclusively that men with high MD and BMI pay more attention to weight-related body areas, as has been found among women with high BMI values [52]. Future studies are needed to confirm and extend these preliminary findings.

This study had some limitations. Several key variables in male body image and muscularity, such as body fat concerns [8], regular physical activity, self-esteem, and the use of anabolic-androgenic steroids and related substances [10], were not properly controlled in this study. These variables can influence concerns on muscularity and, thus, might also affect body-related AB. Furthermore, no screening questionnaires or structured clinical interviews were used to properly assess the presence of muscle dysmorphia, eating disorders, or other mental disorders. Another limitation is related to the BMI formula, since its ability to account for body mass composition (i.e., lean body mass or muscle mass) has been regarded as limited: using the fat free mass index (FFMI) instead of BMI would have provided a better measure for muscle mass [53]. However, certain reliable and accurate fat free mass index measures (e.g., dual-energy X-ray absorptiometry [54]) were impractical for use in the current study. The use of other anthropometric measures such as triceps skinfold or abdominal circumference would also have been more invasive for our participants, since we would have had to literally touch certain body parts to perform the measurements; this might have increased distress levels among our participants and might have affected the results obtained on the VR-ET task. Finally, even though significant group differences were found between men with high and low MD, the use of the median as an “artificial categorization” of a continuous variable might imply some limitations that should be considered: for instance, in terms of the statistical power and accuracy of the relations estimated and in terms of the reduction of the relations observed between variables [55]. On the other hand, other researchers have suggested that the costs of artificial categorization using the median in terms of power are relatively small and are more than offset by the gains that it provides in terms of interpretability [56].

Regarding the VR software, some limitations should also be considered. Despite the avatar sharing the same silhouette and BMI as the participant, the general appearance of the virtual body (e.g., clothes, skin color, etc.) was clearly different. Therefore, it is debatable whether participants truly showed an AB toward their own body, as was the intention, or whether they considered the virtual body as different from their own. However, this limitation is shared by all studies that use 3D computer-generated bodies to assess body image disturbances or body-related AB. In fact, a recent study found that computer-generated bodies yield poorer discrimination and increase errors in body perception research [57]. A possible way to overcome this limitation has been presented in recent VR studies, which allow the reproduction of an exact 3D biometric avatar with all the individual’s features [58]. Accordingly, the use of 3D biometric avatars might notably improve studies on body image and body-related AB that use VR. Finally, the fact that two different VR HMDs were used during the whole assessment task (HTC-VIVE-PRO for inducing FBI and HMD FOVE-ET for measuring gaze) might have reduced FBI levels when participants had to change from one VR HMD to the other. This limitation could have been overcome by using the new generation of VR HMD with advanced ET devices (e.g., HTC VIVE-Pro Eye), which has a complete full-body tracking system and can be used to elicit FBI over the virtual body and measure gaze patterns.

This study might have noteworthy implications for future research and clinical practice involving body image in men. Combining VR and ET technologies to assess AB (e.g., towards body, food, or other stimuli) might lead to new possibilities in the upcoming years. For instance, the use of immersive VR environments might enable the reproduction of real-life environments, such as gyms and dressing rooms, to assess individuals’ gaze patterns towards their own bodies and/or towards other avatars with different body sizes. Additionally, both technologies could be used to implement interventions based on retraining dysfunctional body-related AB. Some studies have already attempted to retrain AB in women with high body dissatisfaction [59,60,61] or in individuals with eating disorders [62], in an approach referred as attentional bias modification training [62].

Considering the similarities between muscle dysmorphia and some eating disorders, not only in their etiology [17,18] but also in their treatment approaches [63], a similar AB modification approach could be also used in men with high MD and/or with muscle dysmorphia diagnosis and as a complement of cognitive behavioral body exposure therapies, for instance, by systematically retraining individuals with MD and/or with muscle dysmorphia to attend equally at their muscular and nonmuscular related body areas. This sort of intervention may be suitable to reduce not only dysfunctional attentional patterns toward the body but also body dissatisfaction, as has been previously found in women with high body dissatisfaction [59,60,61].

## 5. Conclusions

In summary, the current study provides further evidence about the relationship between body-related attention and MD in men. Our results suggest that men with high MD spend more time and look more frequently at muscle-related body areas compared to men with low MD levels. Furthermore, AB towards specific muscle-related body areas was only found among individuals with high MD. Based on the increasing prevalence of body dissatisfaction and body image disorders in men [6,7], future research should focus not only on assessing but also on retraining dysfunctional attentional patterns. The current study used an innovative VR embodiment-based procedure with an ET assessment task that might be especially suitable for future studies aiming to retrain body-related AB in men with high MD and/or with muscle dysmorphia.

## Figures and Tables

**Table 1:** Mean and standard deviation (*SD*) of the eye-tracking measures.

Measures	Men with High MD			Men with Low MD		
	M-AOIs	NM-AOIs	Difference between M-vs. and NM-AOIs	M-AOIs	NM-AOIs	Difference between M-vs. and NM-AOIs
Complete fixation time	14,322 (5127)	8572 (4073)	5750 (8712) **	9651 (4435)	12,039 (5113)	−2387 (8487)
Number of fixations	24.85 (7.69)	15.35 (8.33)	9.50 (11.64) **	18.25 (8.97)	17.25 (6.58)	1.00 (9.74)

Note: Muscle (M)-related and nonmuscle (NM)-related areas of interest (AOIs); MD, muscularity dissatisfaction. ** Follow-up analyses, *p* < 0.01.
